# HALC: High throughput algorithm for long read error correction

**DOI:** 10.1186/s12859-017-1610-3

**Published:** 2017-04-05

**Authors:** Ergude Bao, Lingxiao Lan

**Affiliations:** 1 School of Software Engineering, Beijing Jiaotong University, 3 Shangyuan Residence, Haidian District, Beijing, 100044 China; 2 Department of Botany and Plant Sciences, University of California, Riverside, 900 University Ave., RiversideCA, 92521 USA

**Keywords:** PacBio long reads, Error correction, Throughput

## Abstract

**Background:**

The third generation PacBio SMRT long reads can effectively address the read length issue of the second generation sequencing technology, but contain approximately 15% sequencing errors. Several error correction algorithms have been designed to efficiently reduce the error rate to 1%, but they discard large amounts of uncorrected bases and thus lead to low throughput. This loss of bases could limit the completeness of downstream assemblies and the accuracy of analysis.

**Results:**

Here, we introduce HALC, a high throughput algorithm for long read error correction. HALC aligns the long reads to short read contigs from the same species with a relatively low identity requirement so that a long read region can be aligned to at least one contig region, including its true genome region’s repeats in the contigs sufficiently similar to it (similar repeat based alignment approach). It then constructs a contig graph and, for each long read, references the other long reads’ alignments to find the most accurate alignment and correct it with the aligned contig regions (long read support based validation approach). Even though some long read regions without the true genome regions in the contigs are corrected with their repeats, this approach makes it possible to further refine these long read regions with the initial insufficient short reads and correct the uncorrected regions in between. In our performance tests on *E. coli*, *A. thaliana* and *Maylandia zebra* data sets, HALC was able to obtain 6.7-41.1% higher throughput than the existing algorithms while maintaining comparable accuracy. The HALC corrected long reads can thus result in 11.4-60.7% longer assembled contigs than the existing algorithms.

**Conclusions:**

The HALC software can be downloaded for free from this site: https://github.com/lanl001/halc.

**Electronic supplementary material:**

The online version of this article (doi:10.1186/s12859-017-1610-3) contains supplementary material, which is available to authorized users.

## Background

The Illumina sequencing technology, as a representative of second generation sequencing technology, can produce reads of several hundred bases long (called short reads) with an error rate <1% (dominated by base substitutions) and a cost of approximately $0.03–0.04 per million bases [1]. The low cost of short reads has greatly facilitated the process of sequencing and analyzing new species; however, the limited read length can prohibit sequencing completeness and analysis accuracy. For example, a tremendous number of species have been assembled from short reads, but most of the assemblies are incomplete and fragmented into several thousands of contigs [2, 3]. To address this issue, the PacBio SMRT sequencing technology, as a representative of third generation sequencing technology, has been attracting more and more attention since its commercial release in 2010 [4]. This technology can currently produce reads of 5-15K bases and some of 100K bases (called long reads) with a cost of approximately $0.4-0.8 per million bases [1, 5]. With this technology, it becomes easier to assemble more complete sequences and perform more accurate analyses [6–8]. Depending on how the long reads are used, sequencing projects can be grouped into two classes.

- *Short and long read hybrid sequencing projects* obtain short reads of sufficient coverage as well as long reads of low or moderate coverage from the same species and assemble them together. When the coverage is low, long reads can fill gaps or form scaffolds for the corresponding short read assemblies [9]; when the coverage is moderate, long reads can assemble together with the corresponding short reads [3, 10–12].
- *Long read alone sequencing projects* obtain long reads of high coverage and assemble them alone [7, 13]. These sequencing projects are not as common as the short and long read hybrid sequencing projects because they are more expensive, as the long reads have higher cost than short reads.

Nevertheless, the generated long reads contain 10-15% errors (dominated by insertions and deletions in uniform distribution) [6], so it is important to design efficient algorithms to correct them.

Several error correction algorithms for long reads have been proposed, including PacBioToCA ([6]; the algorithm from the Celera assembler [13]), LSC [8], Proovread [14], CoLoRMap [15], the algorithm from the Cerulean assembler [11], ECTools [16], LoRDEC [17], Jabba [18], DAGCon ([7]; from HGAP assembler), LoRMA [19] and the algorithms from the FALCON and Sprai assemblers (not published). The long read error correction algorithms can be grouped into three classes.

- *Short read based algorithms* PacBioToCA, LSC, Proovread and CoLoRMap align the short reads from the same species to the long reads and use the aligned short reads with low error rate to perform error correction. These algorithms are usually used in short and long read hybrid sequencing projects.
- *Short read assembly based algorithms* the algorithm from Cerulean, ECTools, LoRDEC and Jabba all align the long reads to the de Bruijn graph constructed or contigs assembled from the short reads from the same species to perform error correction. Because of the continuity of the de Bruijn graph or contigs, more error rich regions in the long reads can be aligned and corrected with the de Bruijn graph or contigs. Another benefit of using the de Bruijn graph or contigs is that the alignment of long reads to de Bruijn graph or contigs is much faster than the alignment of short reads to long reads. These algorithms are also usually used in short and long read hybrid sequencing projects.
- *Long read alone algorithms* DAGCon, PacBioToCA in its self-correction mode and the algorithms from FALCON and Sprai find multiple sequence alignments among the long reads, while LoRMA aligns the long reads to the de Bruijn graphs constructed from themselves to perform error correction. These algorithms usually require long read coverage as high as 60–100 × and are thus used in the long read alone sequencing projects.

It is worthwhile to note that there are also many short read error correction algorithms for the second generation sequencing technology [20], but they do not work for long reads due to the different error model. The existing long read error correction algorithms could achieve error rates of approximately 1%, but they must discard a large amount of uncorrected bases and thus lead to low throughput. For example, as listed in [7], PacBioToCA and LSC must discard 42.6-87.1% bases in a human brain long read library to achieve the 1% error rate in the corrected and outputted bases. For another example, in [16] and [17], ECTools and LoRDEC also discard 18.2-70.0% bases in *E. Coli* read libraries for error correction. Such a loss of bases is not economical considering the higher cost of long reads compared to short reads, and it may also reduce the completeness of downstream assemblies and the accuracy of analysis. This point was discussed in [14]: “a decrease in throughput could have a strong impact on the further steps of the projects, especially the assembly”. Also, as reported in [16], with 4.7-24.2% bases discarded, the lengths of assemblies decrease by 14.3-89.0% for the *S. cerevisiae*, *A. thaliana* and *O. sativa* read libraries.

The low throughput discussed above is because of the following two problems.

- *Error richness problem*: some long read regions are error rich, and it is difficult to align them with sufficient identity to the reference data (short reads for short read based algorithms, short read assembly for short read assembly based algorithms or the other long reads for long read alone algorithms) for correction, or it is difficult to validate and distinguish the true alignments from many false ones aligning them with lower identity.
- *Lack of reference data problem*: some long read regions do not have sufficient reference data for correction, due to low read coverage and/or sequencing gaps.

The short read assembly based algorithms could address the error richness problem to some extent by aligning an error rich long read region with relatively low identity requirements, and then validating the candidate alignments and accepting the one that forms a continuous alignment with its adjacent regions’ alignments in the de Bruijn graph or contigs. For example, the algorithm from Cerulean validates long read regions’ alignments to contigs of small lengths by first aligning their adjacent regions to contigs of large lengths and then accepting the former alignments adjacent to the latter in the contigs; LoRDEC and Jabba validate long read regions’ alignments of low identity to the de Bruijn graph by referencing their adjacent regions’ alignments of high identity and then accepting the former alignments adjacent to the latter in the de Bruijn graph. This validation approach is thus called the *adjacent alignment based validation approach*. Some of the remaining algorithms can also address the error richness problem to an extent by making alignments of several passes with different parameter settings [14, 19] or by aligning one pair of paired-end short reads by referencing the alignments of the other pair [15]. However, none of the existing algorithms could address the lack of reference data problem.

To further address the error richness problem and also the lack of reference data problem, in this paper, we propose a novel short read assembly based algorithm called HALC: High throughput Algorithm for Long read error Correction. HALC uses the contigs assembled from the corresponding short reads to correct the long reads. It aligns the long reads to the contigs with a relatively low identity requirement, so that a long read region could be aligned not only to its true genome region but also to the genome region’s repeats in the contigs for correction. This novel alignment approach can address the lack of reference data problem and is called the *similar repeat based alignment approach*. It then validates each long read region’s alignments with the adjacent alignment based validation approach and also by referencing other long read regions’ alignments. This novel validation approach can further address the error richness problem and is called the *long read support based validation approach*.

## Implementation

### Underlining approaches

Below are the details of HALC’s two novel approaches, the similar repeat based alignment approach and the long read support based validation approach, as well as the adjacent alignment based validation approach.

- *Similar repeat based alignment approach* (novel): a long read region could be aligned to its similar repeats in the contigs to guarantee that one long read region is aligned to at least one contig region for correction. Here, a long read region’s *similar repeats* are the genome regions of <15% difference to the long read region’s true genome region [21]. The similar repeats can be located in the contigs by alignment algorithms with dedicated parameter tunings. By this approach, a long read region of approximately 15% error rate compared to its true genome region can be aligned and converted to its similar repeat of <15% difference from the true genome region. The reduced error rate makes it possible to further refine the long read region with the initial short reads and thus reduce the error rate to <1%. It is worth noting that although the existing error correction algorithms for both second and third generation sequencing technologies try to avoid alignments to repeat regions [6, 22], our observation and experimental results, in contrast, demonstrate the possibility to make use of some of the alignments (see the “Discussion” section for details).
- *Long read support based validation approach* (novel): the alignments of a long read region and its adjacent regions in the same long read are validated together, and the ones supported by a sufficient number of adjacent regions from the other long reads are accepted. Here, the alignments of two adjacent long read regions are *supported* by another two adjacent long read regions if the latter are aligned to the same contig regions as the former. With this approach, among several aligned contig regions of a long read region, the one corresponding to its true genome region (if exists) is accepted after validation. The prerequisite of this approach is that different long reads should be aligned to a unified set of contig regions, and one alignment of a long read region is to one contig region in the set; otherwise, it is difficult to check if two adjacent long read regions are aligned to the same contig regions as another two.
- *Adjacent alignment based validation approach* (existing): the alignments of a long read region and its adjacent regions in the same long read are validated together, and the ones aligned adjacent to each other in the contigs are accepted. With this approach, among several candidate alignments of a long read region, the one forming the alignment of the highest continuity is accepted after validation.

**Fig. 1:**
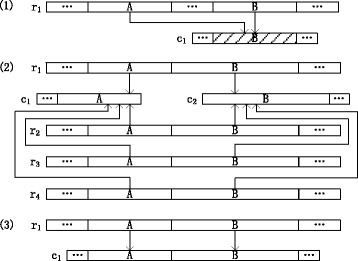
Illustrations of the approaches discussed in the “Background” section. The similar repeat based alignment approach, the long read support based validation approach and the adjacent alignment based validation approach are illustrated in (*1*), (*2*) and (*3*), respectively. (*1*) Long read region *A* in *r*_1_ does not have its true genome region in contig *c*_1_, but it could be aligned to its similar repeat *B* (*shaded*), which is the true genome region of long read region *B* in contig *c*_1_. (*2*) Adjacent long read regions *A* and *B* in *r*_1_ are aligned to contig region *A* in *c*_1_ and contig region *B* in *c*_2_, respectively. These alignments are accepted after validation with a sufficient number of long reads *r*_1_, *r*_2_, *r*_3_ and *r*_4_ supporting them. (*3*) Adjacent long read regions *A* and *B* in *r*_1_ are aligned to the adjacent contig regions *A* and *B* in *c*_1_, respectively, and are thus accepted after validation

Figure 1 illustrates these approaches. Combining these approaches, if a long read region has its true genome region in the contigs, several alignments to the true genome region and its similar repeats are obtained through the similar repeat based alignment approach, and the alignment to the true genome region is accepted using the long read support based validation approach as well as the adjacent alignment based validation approach. The long read region can thus be corrected using the true genome region. Otherwise, if a long read region does not have its true genome region in the contigs, the alignment to a similar repeat is obtained and accepted. The long read region can thus be converted to the similar repeat and then refined using the initial short reads.

### Algorithm overview

The HALC algorithm consists of the following five major steps, with the long reads, the short reads from the same species and the contigs assembled from the short reads as input.

1. Align the long reads to the contigs with a relatively low identity requirement so that a long read region can be aligned to its true genome region or to similar repeats in the contigs.
2. Split the aligned contig regions and the long read regions so that different long reads are aligned to a unified set of contig regions, and one alignment of a long read region is to one contig region in the set.
3. Construct a contig graph from the long read region alignments so that one long read’s alternative alignments can be represented by different paths, and the alignment with the highest long read support and continuity has the minimum total edge weight.
  2.1 Construct a graph representing one aligned contig region as a vertex and representing adjacent long read regions’ alignments to two contig regions as an edge between their vertices.
  2.2 Assign a small weight to the graph edge between two vertices if the long read regions’ alignments are supported by a large number of long read regions, or if the aligned contig regions are adjacent.
4. For each long read, find the paths representing its alternative alignments in the contig graph, and use the one with the minimum total edge weight to correct it.
5. Refine the similar repeat corrected long read regions with the short reads.

**Fig. 2:**
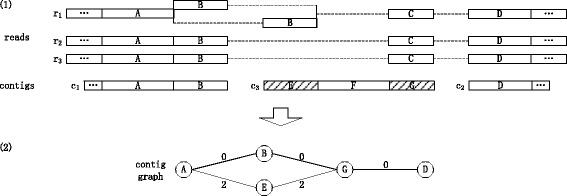
Illustration of the HALC algorithm. Long reads *r*_1_ to *r*_3_ are aligned to contigs *c*_1_ to *c*_3_ with a relatively low identity requirement based on the similar repeat based alignment approach in (*1*), and a contig graph is constructed to validate the alignments and correct the long reads based on the long read support based validation approach and the adjacent alignment based validation approach in (*2*). (*1*) The long read region *r*_1_(*B*) (region *B* of *r*_1_; below follows) is error rich, so it is aligned either to its true genome region in the contigs *c*_1_(*B*) or its similar repeat *c*_3_(*E*) (*shaded*). The reads *r*_1_(*C*), *r*_2_(*C*) and *r*_3_(*C*) do not have their true genome regions in the contigs and thus are aligned to their similar repeat *c*_3_(*G*) (shaded). The aligned contig region *c*_1_(*A* *B*) is split into *c*_1_(*A*) and *c*_1_(*B*), and the long read regions are split accordingly. (*2*) A contig graph is constructed, with vertices *A*, *B*, *D*, *E* and *G* representing the aligned contig regions connected by weighted edges. Edge (*A*,*B*) (edge between vertices *A* and *B*; below follows) is weighted 0, since the contig regions *A* and *B* are adjacent. (*B*,*G*) and (*G*,*D*) are weighted 0, since sufficient adjacent long read regions are aligned to contig regions *B* and *G* and *G* and *D*, respectively. As a result, a path of the minimum total edge weight to correct all the long reads is found containing vertices *A*, *B*, *G* and *D*. The reads *r*_1_(*C*), *r*_2_(*C*) and *r*_3_(*C*) are corrected using their similar repeats and can be refined with the initial short reads

Steps 1 and 5 are based on the similar repeat based alignment approach, step 2 guarantees the prerequisite of the long read support based validation approach, and steps 3-4 are based on the long read support based validation approach and the adjacent alignment based validation approach. It is worth noting that the HALC algorithm does not try to maximize the total identity between a long read and the aligned contig regions because considering the high error rate of the long reads, the long read alignment of the maximum total identity may not be the one to the true genome regions. Step 1 is sufficient to guarantee the identity between a long read region and the aligned contig region. Table 1 shows the correspondence between the steps of the algorithm, the approaches the steps are based on, and the problems addressed by the approaches. Figure 2 illustrates the HALC algorithm.

**Table 1:** Correspondence of algorithm steps, approaches and problems addressed

Steps	Approaches	Problems
1	Similar repeat based alignment	Lack of reference data
3-4	Long read support and adjacent alignment based validation	Error richness
5	Similar repeat based alignment	Lack of reference data

### Long read alignment to contigs

In this step, we align the long reads to the contigs with BLASR [23] because (1) it is specifically designed for long read alignment tolerating large numbers of insertions and deletions, and (2) in our experience, the HALC algorithm showed better performance with BLASR than with several other aligners such as BLAST [24], BLAT [25] and MUMMER [26]. The parameter settings of BLASR are *-bestn 20 -minMatch 8 -nCandidates 30 -maxScore 2000 -minAlnLength 15*, with a trade-off between alignment sensitivity and accuracy so that the long read regions are aligned either to their true genome regions or to similar repeats in the contigs. To further improve the alignment sensitivity, we use scaffolds rather than contigs as input because scaffolds contain additional information about contig orientations and orders, and this information could help guide BLASR alignment. For simplicity, we continue using the term contigs rather than scaffolds in the following discussion.

### Splitting of contig and long read regions

We split the aligned contig regions and the long read regions following the two rules below. In these two rules, an aligned contig region or long read region is denoted by its starting and ending positions in the underlining genome. For example, an aligned contig region *c* starting at genome position *x* and ending at *y* is denoted as *c*(*x*,*y*).

- Two aligned contig regions *c*(*x*,*y*) and *c*(*x* ^′^,*y* ^′^) of the same contig are split into three contig regions *c*(*x*,*x* ^′^), *c*(*x* ^′^,*y*) and *c*(*y*,*y* ^′^), if *x*<*x* ^′^<*y*<*y* ^′^.
- Two long read regions *r*(*x*,*y*) and *r*(*x* ^′^,*y* ^′^) are split into three long read regions *r*(*x*,*x* ^′^), *r*(*x* ^′^,*y*) and *r*(*y*,*y* ^′^), if *r*(*x*,*y*) is aligned to contig regions *c*(*x*,*x* ^′^) and *c*(*x* ^′^,*y*), and *r*(*x* ^′^,*y* ^′^) is aligned to contig regions *c*(*x* ^′^,*y*) and *c*(*y*,*y* ^′^).

These rules are for the general case that two aligned contig regions intersect, while small adjustment can be made to accommodate the case in which one contig region is contained in the other. In practice, long read regions from the same genome region usually contain many differences, so the boundaries of their alignments may be close but different. Therefore, we consider two aligned contig regions *c*(*x*,*y*) and *c*(*x* ^′^,*y* ^′^) (or two long read regions *r*(*x*,*y*) and *r*(*x* ^′^,*y* ^′^)) as the same contig region (or long read region) without further splitting them if |*x*−*x* ^′^|<*δ* and |*y*−*y* ^′^|<*δ*, where *δ* is a small deviation value. The HALC software provides an option *-boundary* to set this value (4 bp by default).

### Graph construction

We construct a contig graph with each vertex per aligned contig region and each edge between two vertices if there is at least one pair of adjacent long read regions aligned to the two contig regions. In most of the cases, different pairs of adjacent long read regions can be aligned to the same two contig regions in the same orientation. More accurately, however, different pairs of adjacent long read regions can be aligned to the same two contig regions in four orientations: forward-forward, forward-reverse, reverse-forward, and reverse-reverse. The contig graph should thus have two vertices for one aligned contig region to represent both the forward and reverse alignments and four edges between the vertices for two aligned contig regions. Therefore, the HALC software provides an option *–accurate* to enable considering the different orientations (yes by default).

### Graph weighting

We weight each edge between two vertices in the graph following the two rules below. The first rule guarantees 0 weight for the edges corresponding to the long read regions’ alignments to adjacent contig regions, and the second rule guarantees small weights for the edges corresponding to the long read regions’ alignments supported by a large number of long read regions.

- If the aligned contig regions of the vertices are adjacent to each other in the initial contig, assign a weight of 0 to the edge.
- If the aligned contig regions are far from each other, assign a weight of *m* *a* *x*{*C*_0_−*C*,0} to the edge, where *C*_0_ is the expected long read coverage on the contigs, and *C* is the number of adjacent long read regions aligned to the two contig regions.

The expected long read coverage *C*_0_ on the contigs can be calculated automatically by checking the average number of long reads covering a contig base, but the HALC software also provides an option *-coverage* for manual input.

### Long read correction

For each long read, we find the paths representing its alternative alignments in the contig graph and use the one with the minimum total edge weight to correct it. To calculate and compare the total edge weight from one vertex representing the first long read region’s aligned contig region to one vertex representing the last long read region’s aligned contig region, dynamic programming is used with the following function:

1 $$ T(i+1, k) = {min}_{j}\{T(i, j) + W_{jk}\}  $$

Here, *T*(*i*,*j*) is the minimum total edge weight from one vertex representing the first long read region’s aligned contig region to the vertex representing the *i*th long read region’s *j*th aligned contig region; *T*(*i*+1,*k*) is the minimum total edge weight from one vertex representing the first long read region’s aligned contig region to the vertex representing the *i*th long read region’s *j*th aligned contig region, and then to the vertex representing the (*i*+1)th long read region’s *k*th aligned contig region; *W*_*jk*_ is the edge weight between the vertex representing the *i*th long read region’s *j*th aligned contig region and the vertex representing the (*i*+1)th long read region’s *k*th aligned contig region. After the path is found, the long read is compared to the list of aligned contig regions in the path and corrected. Adjacent long read regions corrected with distant contig regions are likely to be the ones corrected with similar repeats, so they are recorded for refinement in the next step. There are two things to note in this step. (1) The shortest path algorithms (e.g. Dijkstra’s algorithm) cannot be used to find the path with the minimum total edge weight for a long read because the minimum total edge weight requirement is restricted to the paths representing the long read’s alternative alignments. (2) If there is more than one path with the same minimum total edge weight, the alignment identity is used to break the tie.

### Refinement

We further correct the similar repeat corrected long read regions recorded in the previous step by calling an existing error correction algorithm, LoRDEC. The *k*-mer size of LoRDEC is set to 25, for a trade-off between error correction sensitivity and accuracy. We use LoRDEC rather than implementing the function ourselves because LoRDEC is efficient and accurate in achieving the function [17]. This step can be skipped if very few similar repeats are used to perform error correction. Therefore, HALC provides two modes: an *ordinary* mode and a *repeat-free* mode (ordinary mode by default). In the repeat-free mode, HALC skips this step by filtering very small alignments (< 300 bp) in the graph construction step above and avoiding the use of similar repeats for error correction.

### Software implementation

HALC is implemented in C++ for Linux operating systems. Its input includes the long reads, the short read contigs and the initial short reads, and it outputs the error corrected (1) full long reads, (2) trimmed long reads that do not contain the uncorrected regions in read heads and tails, and (3) split long reads that do not contain the uncorrected regions and very short corrected regions (<100 bp; [17]).

## Results

### Experimental design

To evaluate the performance of HALC, we ran HALC on three data sets from the species, *E. coli*, *A. thaliana* and *Maylandia zebra*, of small, medium and large genome sizes, respectively (see the “Data sets and computing environment” section and Additional file 1: Table S1 for details). The coverage of the long read sets was 11-39x, while the coverage of the short read sets was 35-51x, as HALC is suitable for short and long read hybrid projects, and these levels are the common coverage requirements for this class of projects. SOAPdenovo2 [2] was used to assemble the short reads into contigs. This choice was based on the GAGE evaluation of different assemblers on variable data sets [27]: SOAPdenovo2 and ALLPATHS-LG [3] are among the fastest and most accurate short read assemblers on variable data sets, but the latter’s hard requirement for the mate pair library is usually too stringent for sequencing projects with long reads. For comparison, we also ran PacBioToCA, LSC, Proovread, CoLoRMap, ECTools, LoRDEC and Jabba on the three data sets. The same set of contigs generated by SOAPdenovo2 was used for ECTools. Cerulean was not run because it does not directly output the error corrected long reads. DAGCon, PacBioToCA in self-correction mode and the algorithms from FALCON and Sprai were not compared because they are all for long read alone sequencing projects. We measured the correction completeness and accuracy of the compared algorithms by aligning the error corrected and initial long reads to their corresponding genomes (see the “Performance measurements” section for details).

Furthermore, to see the effect of error correction upon the final assemblies with both short and long reads, we assembled the short read contigs and the long reads with or without error correction together using the SPAdes assembler, which is fast and accurate for long sequence assemblies [10]. We also measured the completeness and accuracy of the obtained contigs by aligning them to the corresponding genomes (see the “Performance measurements” section for details). It is worth noting that the main purpose of this paper is not to compare assembly performance downstream of error correction algorithms, so this test is greatly simplified.

In addition, to see HALC’s performance on transcriptomic data, we also compared HALC with the existing algorithms on a transcriptomic data set from *S. cerevisiae* and made measurements by aligning the error corrected and initial long reads to the corresponding transcriptome (see the “Data sets and computing environment” section and the “Performance measurements” section for details). Trinity was used to assemble the transcriptomic short reads, also because of its good performance [28].

Finally, we tested HALC by varying the short read assemblers on the *E. coli* data set and the *S. cerevisiae* data set. For the former data set, we also assembled the short reads using other typical assemblers, Velvet [29] and ABySS [30], and for the latter, we also assembled the short reads by other typical transcriptome assemblers, Oases [31] and Trans-ABySS [32]. We then ran HALC with the assemblies and performed the same measurements as above.

All of the software or algorithms above were used with the default settings. Only the corrected split long reads were compared and assembled in these tests, so the results could not be affected by the uncorrected bases. The split long reads for LSC were obtained by filtering bases with short read coverage ≤2.

### Data sets and computing environment

The long reads of *E. coli*, *A. thaliana*, *Maylandia zebra* and *S. cerevisiae* were from the PacBio DevNet site, NCBI accession SRX533608, NCBI accession SRX985423, and NCBI accessions SRR2102571 and SRR2102572, respectively. The corresponding short reads were from NCBI accession ERR022075, NCBI accession ERR469286, NCBI accession SRX033046, and NCBI accession SRR059177, respectively. The genomes of *E. coli* and *A. thaliana* were from NCBI accession NC_000913 and the TAIR FTP site, respectively. The genome of *Maylandia zebra* was not available, so the recently improved scaffolds were downloaded from NCBI accession GCF_000238955.2 to approximate the genome [33]. The transcriptome of *S. cerevisiae* was from the Ensembl FTP site. Details of the data sets are listed in Additional file 1: Table S1. All experiments were performed in a computing node of a computer cluster with 16 cores of 2.3 GHz and 512 GB memory, and the numbers of processes and threads allocated for the algorithms are listed in Additional file 1: Table S2.

### Performance measurements

We aligned the genomic long reads to the corresponding genomes to evaluate their quality. The BWA-MEM aligner was used for these alignments because it is a typical aligner for genomic sequences with fast speed and high sensitivity [34]. We made the following measurements: (1) *throughput (TH)* is the number of corrected and outputted bases over the total number of initial long read bases (throughput is 100% for the initial long reads); (2) *alignment ratio* is the number of aligned bases over the total number of outputted bases; (3) *alignment identity* is the identity of the aligned bases; (4) *genome fraction* is the number of genome bases covered by the long reads over the total number of genome bases; (5) *number of reads*; (6) *average read length*.

Referencing the Error Correction Evaluation Toolkit for short reads [20] and for full long reads [17], we also implemented a version for the split long reads and obtained, in the outputted bases, the number of corrected errors (true positive or *TP*), the number of falsely converted correct bases (false positive or *FP*), the number of uncorrected errors (false negative or *FN*), and the number of unconverted correct bases (true negative or *TN*). With these numbers, due to the errors’ uniform distribution in the long reads, we can estimate the total number of errors in the initial long reads as the number of errors in the outputted bases over the throughput, i.e. $EI = \frac {TP + FN}{TH}$. We can also estimate the total number of correct bases in the initial long reads as the number of correct bases in the outputted bases over the throughput, i.e. $CI = \frac {TN + FP}{TH}$, and thus the number of correct bases in the discarded bases as the total number of correct bases in the initial long reads minus the number of correct bases in the outputted bases, i.e. *C* *D*=*C* *I*−(*T* *N*+*F* *P*). Therefore, we made the following measurements: (7) *sensitivity* is calculated as $\frac {TP}{EI}$; (8) *specificity* is calculated as $\frac {TN + CD}{CI}$; (9) *gain* is the number of errors effectively corrected without introducing new ones over the total number of errors in the initial long reads, calculated as $\frac {TP - FP}{EI}$. It is worth noting that our Error Correction Evaluation Toolkit requires the correspondence information between a split long read and its initial long read, so it does not work for PacBioToCA, which does not provide this information in the output.

In addition, we aligned the contigs assembled with the long reads to the corresponding genomes to evaluate the impact of error correction on the final assemblies. Following [27] and using the QUAST toolkit [35], we split a contig into two subcontigs if the subcontigs were aligned at least 1K bp apart indicating a misassembly, and then we made the following measurements: (10) *number of contigs* is the number of initial contigs without splitting; (11) *N50* is the split contig size at 50% of the total number of contig bases; (12) *largest contig length* is the largest length of split contigs; (13) *number of covered bases* is the number of genome bases covered by the split contigs; (14) *EPKB* is the number of errors (including misassemblies and indels) per 100K bp in the initial contigs.

In the transcriptomic data set, we aligned long reads to the corresponding transcriptome to evaluate their quality. The BLAT aligner was used to make these alignments because it is a typical aligner for transcriptomic sequences with high sensitivity [25]. We obtained the measurements (1)-(3) above as well as the following measurement: (15) *transcriptome fraction* is the number of transcriptome bases covered by the long reads over the total number of transcriptome bases.

### Results on error correction performance

The performance test results on the *E. coli* data set are listed in Table 2(a). A total of 50.4% bases of the initial long reads can be aligned to the corresponding genome with 95.2% identity, indicating a high error rate in the uncorrected long reads. The existing error correction algorithms PacBioToCA, LSC, Proovread, ECTools and LoRDEC can correct and output 23.5-60.8% of the bases. HALC can obtain 7.2–41.1% higher throughput than PacBioToCA, LSC, Proovread and ECTools and is comparable (<5% difference) to LoRDEC. The alignment ratio, alignment identity and genome fraction of all the algorithms are almost 100% and thus comparable. Except for PacBioToCA and LSC, the average read length of all the algorithms is inversely proportional to the throughput because more but shorter reads can be obtained with higher throughput. The sensitivity and gain of all the algorithms are proportional to the throughput, while the specificity remains comparable.

**Table 2:** Evaluation of error correction performance

Method	Throughput	Alignment ratio	Alignment identity	Genome fraction	N reads	Average read length	Sensitivity	Gain	Specificity
*(a) Long reads of E. coli*									
Initial	100.0%	50.4%	95.2%	100.0%	75152	2381	-	-	-
PacBioToCA^a^	24.2%	100.0%	100.0%	99.5%	53447	810	-	-	-
LSC	53.5%	98.7%	99.9%	99.7%	115960	825	52.6%	51.7%	99.9%
Proovread	57.4%	100.0%	99.9%	99.7%	44986	2284	57.4%	56.8%	99.9%
CoLoRMap	42.8%	99.7%	100.0%	99.9%	70582	1084	42.7%	42.2%	99.9%
ECTools	23.5%	99.9%	99.2%	99.4%	8095	5211	23.4%	21.8%	99.8%
LoRDEC	60.8%	97.8%	100.0%	99.8%	70164	1549	60.7%	60.5%	100.0%
Jabba	52.8%	99.6%	100.0%	98.6%	26459	3568	52.8%	52.7%	100.0%
HALC	64.6%	98.6%	99.9%	99.8%	78731	1467	64.4%	64.0%	99.9%
*(b) Long reads of A. thaliana*									
Initial	100.0%	32.4%	92.4%	82.4%	490418	2645	-	-	-
PacBioToCA^a^	10.7%	99.2%	99.7%	63.9%	260834	535	-	-	-
LSC	25.9%	100.0%	99.5%	71.4%	659123	509	24.2%	22.3%	99.7%
Proovread	27.8%	99.8%	99.7%	79.8%	125786	2864	26.5%	24.9%	99.7%
CoLoRMap	21.4%	99.4%	99.7%	69.3%	230933	1203	20.5%	19.2%	99.8%
ECTools	11.3%	99.8%	99.5%	63.1%	21354	6886	10.8%	9.8%	99.8%
LoRDEC	28.0%	86.4%	99.5%	74.4%	847963	428	25.9%	22.8%	99.6%
Jabba	10.8%	99.6%	99.7%	56.1%	51353	2726	10.5%	9.9%	99.9%
HALC	34.7%	96.5%	99.5%	85.8%	548872	819	33.2%	29.7%	99.3%
*(c) Long reads of Maylandia zebra*									
Initial	100.0%	46.9%	91.3%	91.9%	1307812	10082	-	-	-
LoRDEC	33.6%	97.9%	99.7%	89.5%	7372455	601	32.4%	29.8%	99.6%
HALC	41.2%	98.7%	99.6%	90.7%	4833536	1123	40.2%	37.5%	99.4%

The long reads of tests (a)-(c) are from *E.coli*, *A. thaliana* and *Maylandia zebra*, respectively. The initial and error corrected long reads by PacBioToCA, LSC, Proovread, CoLoRMap, ECTools, LoRDEC, Jabba and HALC are compared in the tests. The performance measurements are listed in the “Performance measurements” section.

^a^Some measurements are not available without the correspondence information between a split long read and its initial long read

The performance test results for the *A. thaliana* data set are listed in Table 2(b). HALC can obtain 6.7-24.0% higher throughput than all the existing algorithms. The performance test results on the *Maylandia zebra* data set are listed in Table 2(c). HALC can obtain 7.6% higher throughput than LoRDEC. In both tests, the alignment ratio, alignment identity, genome fraction, sensitivity, gain and specificity of HALC are comparable to or higher than the existing algorithms, and the average read length of HALC is moderate. The results of PaBioToCA, LSC, Proovread, CoLoRMap, ECTools and Jabba are not shown in Table 2(c) because of their very long running time.

The test results in this section indicate that HALC is efficient in correcting and outputting more bases in the initial long reads than the existing algorithms while maintaining sufficient accuracy.

### Results on long read assemblies

The assembly results for the error corrected *A. thaliana* long reads are listed in Table 3(a). The number of assembled contigs with HALC corrected long reads is 5.8-29.6% smaller than with most of the existing algorithms, and the N50 value, the largest contig length and the number of covered bases with HALC corrected long reads are 11.4-60.7, 26.6-238.5 and 6.1-141.7% larger than with most of the existing algorithms, respectively. The EPKB value with HALC corrected long reads is 6.8-17.4% smaller than with most of the existing algorithms. Generally, the assembly quality is proportional to the throughput of the algorithms, except for ECTools, with much larger read lengths, and LSC and LoRDEC, with relatively smaller read lengths. The assembly results for the error corrected *Maylandia zebra* long reads are listed in Table 3(b). Even though the number of assembled contigs with HALC corrected long reads is 10.6% larger than with LoRDEC, the N50 value, the largest contig length and the number of covered bases with HALC are 35.9, 33.7 and 58.6% larger than with LoRDEC, respectively. The EPKB value with HALC corrected long reads is 23.6% smaller than with LoRDEC. The results with the initial uncorrected long reads are not shown because of the limited assembly quality. The assembly results with the *E. coli* long reads are not shown because almost perfect contigs were obtained with the variable long reads, and there is not much difference. These results indicate that HALC corrected long reads can result in more complete assemblies than the existing algorithms with sufficient accuracy.

**Table 3:** Evaluation of long read assemblies

Method	N Contigs	N50	Largest contig length	N Covered bases	EPKB
*(a) Contigs of A. thaliana*					
PacBioToCA	1629	27806	110971	51672726	119.4
LSC	1284	29305	105354	40390383	128.6
Proovread	1193	37828	233854	50379300	123.9
CoLoRMap	1324	35477	150127	52913748	113.1
ECTools	1218	40122	182238	56377176	143.5
LoRDEC	1331	28133	104370	40034274	145.1
Jabba	618	29548	87500	22681404	102.1
HALC	1147	44684	296154	54821730	119.9
*(b) Contigs of Maylandia zebra*					
LoRDEC	37460	8878	115008	204752772	121.6
HALC	41434	12062	153787	324763656	92.9

The contigs of tests (a)-(b) are for *A. thaliana* and *Maylandia zebra*, respectively. The contigs assembled from the error corrected long reads by PacBioToCA, LSC, Proovread, CoLoRMap, ECTools, LoRDEC, Jabba and HALC are compared in the tests. The performance measurements are listed in the “Performance measurements” section

### Results on transcriptome data

For the transcriptome data, the performance test results on the *S. cerevisiae* data set are listed in Table 4. A total of 7.0% bases of the initial long reads can be aligned to the corresponding transcriptome with 99.5% identity, indicating a high error rate in the uncorrected long reads. The existing error correction algorithms LSC, CoLoRMap, LoRDEC and Jabba can obtain 26.0-33.7% throughput, 14.9-29.8% alignment ratio, 99.8-100.0% alignment identity and 15.5-20.6% transcriptome fraction. HALC can obtain 16.1-23.8% higher throughput, 0.7-15.6% higher alignment ratio, and 0.8-5.9% higher transcriptome fraction than all the existing algorithms with comparable alignment identity. The results of the PacBioToCA, Proovread and ECTools are not shown in Table 4 because of their limited performance. It is worth noting that even though some algorithms, such as Proovread, can achieve much better performance by using tailor-made parameters [14], we did not use this procedure to allow a fair comparison. The test results in this section indicate that HALC is also efficient in correcting transcriptome data.

**Table 4:** Evaluation of error correction performance on the transcriptomic data set of *S. cerevisiae*

Method	Throughput	Alignment ratio	Alignment identity	Transcriptome fraction
Initial	100.0%	7.0%	99.5%	17.0%
LSC	31.2%	20.2%	99.8%	16.5%
CoLoRMap	26.0%	29.8%	99.8%	20.6%
LoRDEC	33.2%	18.8%	99.9%	16.6%
Jabba	33.7%	14.9%	100.0%	15.5%
HALC	49.8%	30.5%	99.4%	21.4%

The initial and the error corrected long reads by LSC, CoLoRMap, LoRDEC, Jabba and HALC are compared. The performance measurements are listed in the “Performance measurements” section

### Results with various short read assemblers

With various short read assemblers, HALC exhibits stable results on the *E. coli* and the *S. cerevisiae* data sets, and the difference for all the measurements is below 5% (data not shown). This result indicates that HALC is not very dependent on the upstream short read assemblers and can be used together with various assemblers and for different data types.

### Running time and memory usage

The running time of HALC on the *E. coli*, A. thaliana, *Maylandia zebra* and *S. cerevisiae* data sets is 0.7h, 7.0h, 53.1h and 0.5h, respectively. The memory usage is 12.3GB, 41.2GB, 33.7GB and 25.9GB, respectively, including the running time and memory usage for short read assemblies by SOAPdenovo2. Compared to the existing algorithms, HALC’s running time is much shorter than for PacBioToCA, LSC, Proovread and ECTools and is comparable to or greater than the running time for LoRDEC. Jabba’s running time is dependent on the genome sizes. Comparatively, HALC’s running time is much smaller on the *Maylandia zebra* data set of large genome size and is larger on the other data sets of small and medium genome sizes. Details of the running time and memory usage are listed in Additional file 1: Table S2. These results indicate that although the main purpose of HALC is to guarantee sufficiently high throughput, it is efficient in running time with acceptable memory usage and can thus scale well for variable project sizes.

### Discussion

The most important concern regarding the HALC algorithm is whether the similar repeat based alignment approach introduces false corrections. In theory, false corrections are possible because after a long region is corrected with its similar repeat, it might be refined with the short reads from the similar repeat instead of the ones from the true genome region. However, this problem is not frequent because the refinement algorithm LoRDEC aligns short reads to a long read region by considering not only the long read region’s identity but also its adjacent regions in the same long read.

Experimentally, if a similar repeat corrected long read region is a false correction not further refined with the short reads from the true genome region, it will be aligned to its similar repeat in the corresponding genome instead of its true genome region. In other words, it will be aligned to a genome region included in the short read contigs instead of the genome region not included in the contigs. Therefore, we refer to the short read contigs to check for false corrections. We aligned both the HALC corrected long reads and the short read contigs to the genomes on the *E. coli*, *A. thaliana* and *Maylandia zebra* data sets used above and calculated the percentage of genome bases not covered by the contigs and the percentage of long read bases aligned to these genome bases. A much larger value of the former than the latter would indicate that many long read regions are aligned to their similar repeats instead of the true genome regions and are thus false corrections. For the *E. coli* data set, the two values are 4.4 and 3.9%; for the *A. thaliana* data set, the two values are 21.5 and 23.6%; for the *Maylandia zebra* data set, the two values are 42.8 and 39.3%, all comparable. This result indicates limited false corrections by HALC. Furthermore, since many similar repeat corrected long read regions are not false corrections and are aligned to the genome regions not included in the contigs, we calculated the identity between the genome bases not covered by the contigs and the long read bases aligned to these bases to see the accuracy of the similar repeat corrected long read regions. The identity values are 99.9, 99.3 and 99.6% for the three data sets, respectively. This result indicates high accuracy of the similar repeat corrected long read regions.

**Fig. 3:**
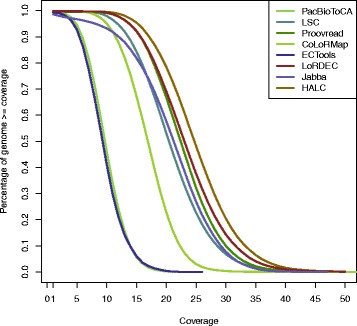
Percentage of genome covered above various long read coverages on the *E. coli* data. The percentage of genome bases (*y*-axis) is plotted with long read coverage from 1 × to 50 × (*x*-axis), corresponding to the error correction results of different algorithms in Table 2(a)

In addition, we also refer to the error corrected long reads produced by the existing algorithms to check for false corrections. We aligned the error corrected long reads by various algorithms to the genomes on all three data sets and calculated the percentage of genome bases above the various long read coverages. A much smaller percentage of genome bases above the small long read coverages for HALC than the existing algorithms, or a much larger percentage of genome bases above the large long read coverages for HALC, would indicate that many long read regions are aligned to their similar repeats instead of the true genome regions and are thus false corrections. The plot for the *E. coli* data set is shown in Fig. 3, and the curve of HALC exhibits a similar switch to the existing algorithms from high coverage to low at 25 ×. The plots of the *A. thaliana* and the *Maylandia zebra* data sets are also available in Additional file 1: Figures S1 and S2, respectively, and similar results can be observed. This result also indicates limited false corrections by HALC. Indeed, the amount of errors contained in the long read assemblies with HALC is another reflection of the false corrections. Table 3 shows that the EPKB values with HALC are smaller or comparable to the ones with most of the existing algorithms, also indicating limited false corrections by HALC (see the “Results on long read assemblies” section).

## Conclusions

This study introduces HALC, a high throughput algorithm for PacBio long read error correction. With the similar repeat based alignment approach, the long read regions without true genome regions in the contigs can be aligned; with the long read support based approach, the long read regions’ alignments with the highest long read support and continuity can get accepted. Hence, more long read bases can be corrected with accuracy. The experimental results indicate that HALC can correct more bases in the long reads than the existing error correction algorithms while achieving comparable or higher accuracy. As a result, HALC can help to obtain more complete assemblies by providing the error corrected long reads.

## Availability and requirements

**Project name:** HALC

**Project home page:** https://github.com/lanl001/halc

**Operating system(s):** Linux

**Programming language:** C++

**Other requirements:** BLASR and LoRDEC

**License:** Artistic License 2.0

**PacBio DevNet site:** https://github.com/PacificBiosciences/DevNet/wiki/E.-coli-Bacterial-Assembly

## Additional file

Additional file 1
**Figure S1.** Percentage of genome above various long read coverages on the *A. thaliana* data. **Figure S2.** Percentage of genome above various long read coverages on the *Maylandia zebra* data. **Table S1.** Data sets used in the evaluation. **Table S2.** Running time and memory usage in the evaluation of error correction performance. (PDF 85.9 kb)

## Abbreviations

CI: Number of correct bases in the initial long reads
CD: Number of correct bases in the discarded bases
EI: Number of errors in the initial long reads
EPKB: Number of errors per 100K bp in the initial contigs
FN: False negative
FP: False positive
TH: Throughput
TN: True negative
TP: True positive
