# Supplementary material for: HALC: High throughput algorithm for long read error correction
Source: BMC Bioinformatics. 2017 Apr 5;18:204. doi: 10.1186/s12859-017-1610-3 (PMC5382505; doi:10.1186/s12859-017-1610-3)
Supplement: Additional file 1 — Figure S1. Percentage of genome above various long read coverages on the A. thaliana data. Figure S2. Percentage of genome above various long read coverages on the Maylandia zebra data. Table S1. Data sets used in the evaluation. Table S2. Running time and memory usage in the evaluation of error correction performance. (PDF 85.9 kb) [file 12859_2017_1610_MOESM1_ESM.pdf]

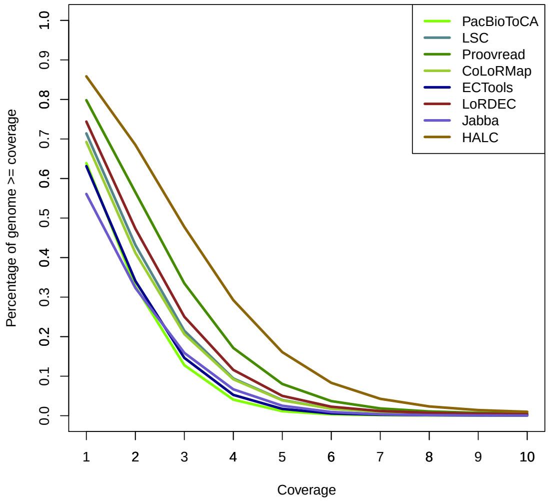

Fig. S1 Percentage of genome above various long read coverages on the *A. thaliana* data. The percentage of genome bases (y-axis) is plotted with long read coverage from 1x to 10x (x-axis), corresponding to the error correction results by different algorithms in Table 2(b).

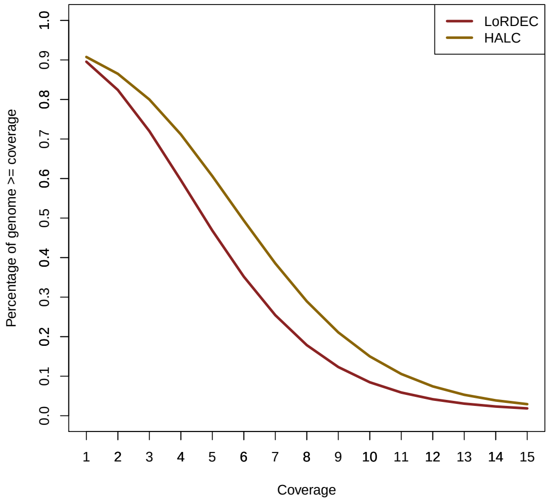

Fig. S2 Percentage of genome above various long read coverages on the *Maylandia zebra* data. The percentage of genome bases (y-axis) is plotted with long read coverage from 1x to 15x (x-axis), corresponding to the error correction results by different algorithms in Table 2(c).

Table S1 Data sets used in the evaluation.

		E. coli	*A. thaliana*	*Maylandia Zebra*	*S. cerevisiae*
Genome/Transcriptome	Accession	NC_000913	TAIR	GCF_000238955.2[1]	Ensembl
	Number of sequences	1	5	3555	6713
	Size	4.6M	119.1M	859.8M	9.1M
Long reads	Accession	DevNet[2]	SRX533608: SRR1284083-SRR1284086	SRX985423: SRR1960950-SRR2126010	SRR2102571-SRR2102572
	Number of reads	75152	490418	1307812	199540
	Average length	2381	2645	10082	145
	Number of bases	179.0M	1297.6M	13186.6M	43.5M
	Coverage	39x	11x	15x	5x
Short reads	Accession	ERR022075[3]	ERR469286	SRX033046: SRR077286-SRR077292	SRR059177[3]
	Number of reads	2.3M	50.5M	298.4M	9.9M
	Read length	101	90	101	76
	Insert length	600	451	101	240
	Number of bases	234.0M	4544.3M	30138.0M	1504.8M
	Coverage	51x	38x	35x	165x

[1] Scaffolds due to lack of complete genome

[2] https://github.com/PacificBiosciences/DevNet/wiki/E.-coli-Bacterial-Assembly

[3] Subset of the accession

Table S2 Running time and memory usage in the evaluation of error correction performance.

Method	N Processes	N Threads	Running time (h)	Memory usage (GB)
(a) On E. coli				
PacBioToCA	1	16	15.3	9.3
LSC	16	1	5.6	14.0
Proovread	4	4	16.3	4.4
CoLoRMap	1	16	5.8	6.5
ECTools	16	1	0.1[1]+0.5	$max\{3.9^1, 2.1\}$
LoRDEC	1	16	0.6	0.8
Jabba	1	16	0.1+0.1	$max\{4.6, 0.5\}$
HALC	1	16	0.1+0.6	$max\{3.9, 12.3\}$
(b) On A. thaliana				
PacBioToCA	1	16	152.0	14.2
LSC	16	1	184.6	14.6
Proovread	4	4	235.2	2.1
CoLoRMap	1	16	181.6	36.3
ECTools	16	1	0.6+182.2	$max\{9.9, 2.1\}$
LoRDEC	1	16	6.2	4.4
Jabba	1	16	0.9+3.0	$max\{100.0, 13.8\}$
HALC	1	16	0.6+6.4	$max\{9.9, 41.2\}$
(c) On Maylandia zebra				
LoRDEC	1	16	21.6	14.1
HALC	1	16	12.7+40.4	$max\{33.7, 16.8\}$
(d) On S. cerevisiae				
LSC	16	1	1.6	1.5
CoLoRMap	1	16	5.6	9.4
LoRDEC	1	16	0.1	2.0
Jabba	1	16	0.4+0.1	$max\{30.1, 0.5\}$
HALC	1	16	1.3+0.4	$max\{15.1, 25.9\}$

[1] Additional time and memory of short read assemblies performed by SOAPdenovo2 for ECTools and HALC, or of preprocessing by Karect and Brownie for Jabba.
